# H-plane gap-RGW horn antenna with very low side lobe level

**DOI:** 10.1038/s41598-024-69376-6

**Published:** 2024-08-07

**Authors:** Mohammad Mohammadpour, Farzad Mohajeri, Seyed Ali Razavi Parizi

**Affiliations:** 1 School of Electrical and Computer Engineering, Shiraz University, Shiraz, Iran; 2 School of Electrical and Computer Engineering, Graduate University of Advanced Technology, Kerman, Iran

**Keywords:** Engineering, Electrical and electronic engineering

## Abstract

This paper presents and applies a new concept of gap-RGW to develop a new configuration for RGW H-plane horn antennas. The proposed antenna is fully metallic and can generate a very low side-lobe level along with a flat gain response over a reasonable bandwidth, which is one of the advantages of this antenna. A prototype of the presented structure is designed and fabricated which provides the impedance bandwidth (S11 < − 10 dB) of about 25% with SLL < − 20 dB and 2 dB gain variation over the operating bandwidth.

## Introduction

Antennas with fan beam radiation patterns have been widely used in different applications such as the Direction of Arrival (DoA) detection radars^1–3^, aircraft landing systems^4,5^, mm-wave imaging^6,7^, and fixed access points. In some practical applications like DoA radars and Local-to-Multipoint Distribution Service (LMDS) systems^8^, fan beam radiation along with very low side lobe level (SLL) over the whole operating bandwidth is of great interest. Using array antennas to achieve low SLL is a common approach but with quite a complicated feeding network. H-plane sectoral horn, as a simple and fully metallic structure, has the potential to perform as a highly-efficient fan beam radiator. Furthermore, the H-plane horn presents a low profile and flat geometry making it useful in systems with space limitations. The main superiority of the horn over the array solution is lower complexity and larger bandwidth^9^. However, in arrays, it is easier to deal with SLL compared to the conventional H-plane horns. So, in this paper, the main goal is to develop a fully metallic H-plane horn with a very low SLL of below − 20 dB over its whole operating bandwidth. With conventional metal H-plane horns, we can get low SLL; however conventional waveguides suffer from fabrication cost and complexity at mm-wave ranges.

Gap waveguide (GW)^10,11^, as a contactless structure with no dielectric loss, has been introduced as an alternative to conventional and substrate integrated waveguides^12–14^. Different types of GW including ridge gap waveguide (RGW), groove gap waveguide (GGW), and microstrip gap waveguide (MGW)^15^, have been used to design and develop various microwave components and particularly fan beam radiating antennas^16–27^. In^16^ the RGW technology is used to develop an H-plane horn antenna with an outer transition at its aperture, resulting in − 15 dB < SLL < − 10 dB. In^17^, an RGW H-plane horn lens based on glide symmetry with an SLL of below − 10.7 dB is presented. In^19^ a GGW H-plane horn with an SLL of below − 16 dB is introduced. The concept of a 2D RGW offset reflector is applied in^20^ to provide fan beam radiation with − 20 < SLL < − 15 dB. In^21^, a semi-circular dielectric-loaded GGW horn is introduced with a maximum SLL of − 10 dB over the operating bandwidth. In^22^ partially dielectric filled RGW and GGW H-plane horns are introduced and the SLL of below − 24 dB and below − 30 dB is reported respectively. In^23^ a single layer symmetrical pillbox antenna with SLL < − 10 dB is presented. In^24^ a dual RGW-based antenna with multiple radiation capabilities is reported which offers a maximum SLL of − 8 dB. We see that except for the structure introduced in^22^, all presented GW-based H-plane antennas give the SLL of above − 20 dB over their operating bandwidth. The structure that we have introduced in^22^ uses dielectric in metal RGW and GGW configurations which enhances the fabrication complexity as well as the ohmic loss.

In this paper, a proof of concept for a new fully metallic GW-based H-plane horn with an SLL of below − 20 dB over the whole operating bandwidth is developed. The proposed structure is a TEM-based horn which is implemented making use of RGW technology. In the proposed structure, the amplitude taper across the horn aperture is improved making use of the new gap-RGW technique which is introduced in this paper for the first time. As the proposed structure is dielectric free, it introduces lower ohmic loss and fabrication complexity compared to the work presented in^22^. The designed sample antenna provides 25% impedance bandwidth (12.5–16.2 GHz) along with an SLL of below − 20 dB and a gain of 8.1–10.7 dBi over the operating bandwidth.

In the following, the topology of the proposed structure is presented. Then, the new gap-RGW concept is introduced and applied to control the amplitude taper across an H-plane horn aperture and consequently reduces its SLL. After that, the simulation and measurement results of the designed prototype are presented and discussed, and finally, concluding remarks are presented.

## Antenna topology

**Figure 1:**
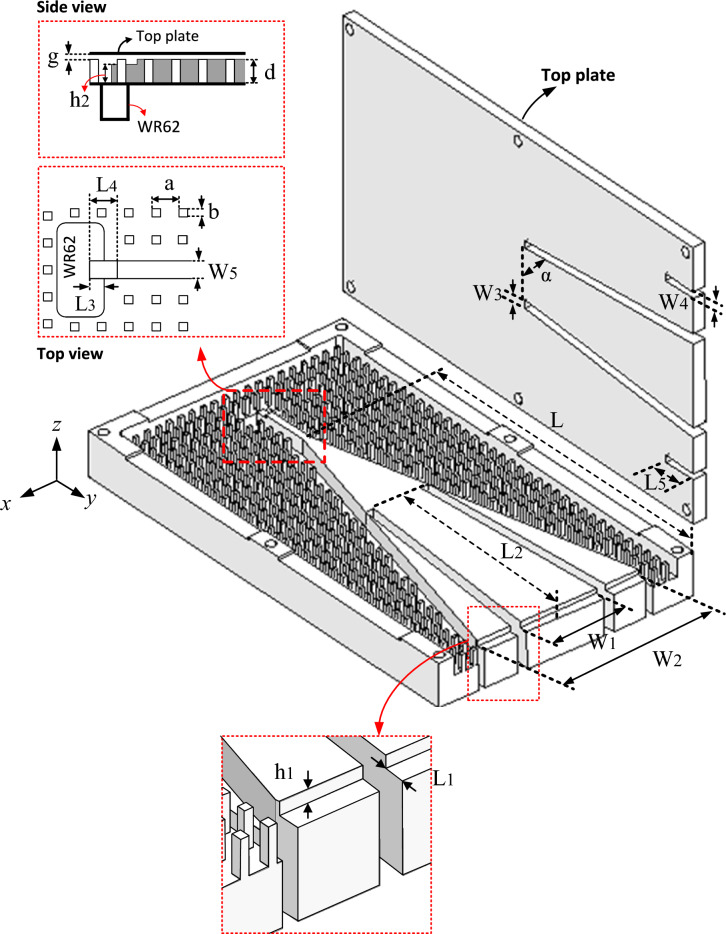
The proposed antenna geometry.

**Figure 2:**
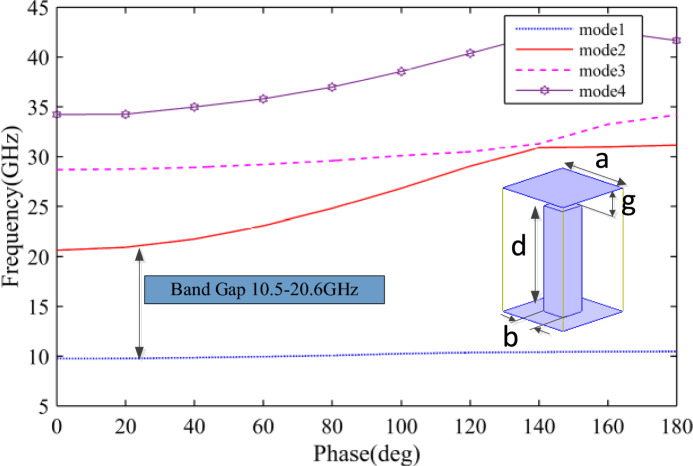
Dispersion diagram of the designed unit cell in an infinite environment.

The proposed antenna topology is shown in Fig. 1. We see an RGW H-plane horn, the same as that presented in^16^, in which a flared ridge is surrounded by a bed of periodic nails and topped by a metal lid with an air gap “g”. Each pin has a square cross-section with the width and the height of “b” and “d” respectively. The pins period is also shown as “a”. By properly choosing the parameters “g”, “d”, “a” and “b” according to the design rules of “Fakir bed of nails”^15^, a stop band for parallel plate modes can be provided over the desired frequency range. Here, the dimensions of the pre-mentioned geometrical parameters are adopted to provide a stop band over the whole Ku band (12–18 GHz). In Fig. 2, the dispersion diagram of the designed pin surface is depicted and we see that a stop band over the frequency range of 10.5–20.6 GHz is provided covering the entire desired bandwidth.

The dimensions of the flared RGW, “L” and “W2”, can be initially selected according to the well-known design rules of conventional H-plane horn antennas. However, then fine-tuning may be needed to maximize the phase uniformity across the horn aperture. Similar to^16^, the RGW end at the horn aperture is loaded by a stepped discontinuity, with geometrical parameters “h1” and “L1”, to improve the impedance matching between the horn aperture and the outer space. In fact, the whole configuration of the proposed structure is similar to that presented in^16^, however here a few gaps are introduced to the ridge and upper plate.

In Fig. 1, we see that two pairs of long longitudinal gaps with the width of “W3” are introduced to both the flared ridge and its top plate. These two pairs of gaps are congruous and exactly aligned with each other. In each pair, the two gaps are symmetrical; each is flared by “α” relative to the x-axis (see Fig. 1). There is also a pair of short longitudinal gaps of the length “L5” and the width “W4” which are located at the top plate. The mission of the introduced longitudinal gaps is to improve SLL by proper control of the amplitude taper across the horn aperture which is fully discussed in the next section.

To facilitate the measurement process, the proposed antenna is fed by a standard WR62 waveguide as shown in Fig. 1. A transition between WR62 and RGW feeding line is realized by loading the RGW line with two stepped discontinuities making it possible to provide proper impedance matching. The feeding line width “W5” along with the transition geometrical parameters “L3”, “L4” and “h2” are selected so that the RGW feed line is well impedance matched with WR62.

## Gap-RGW

### Concept

**Figure 3:**
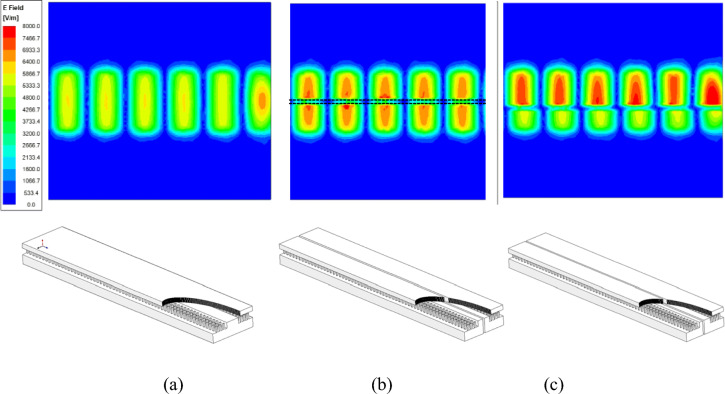
The simulated field distribution inside (**a**) ordinary RGW (**b**) central symmetric gap-RGW (**c**) asymmetric gap-RGW.

The new concept of gap-RGW and its principle of operation, as shown in Fig. 3, is first presented in here. We see that in gap-RGW, a longitudinal gap is introduced to the RGW wave path by carving both the ridge and the top plate simultaneously. If the width of the introduced longitudinal gap is small enough then no leakage is occurred through it as it does not cut the surface currents associated with the TEM wave propagating inside RGW.

**Figure 4:**
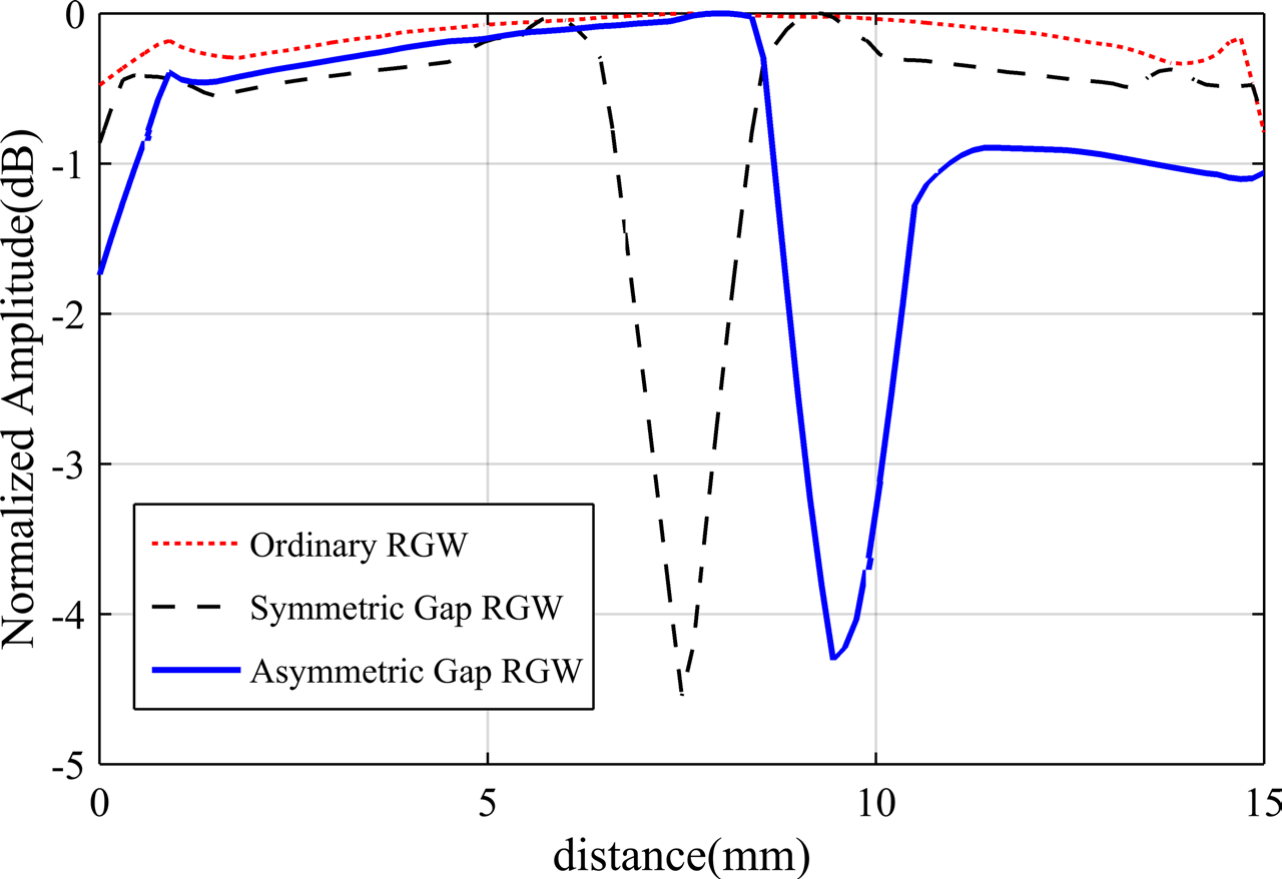
The simulated E-field amplitude distribution over the cross section of ordinary RGW, symmetric gap-RGW, and asymmetric gap-RGW.

In Fig. 3, the wave propagation inside a gap-RGW for the two cases of central symmetric and asymmetric geometries is illustrated and compared with that of an ordinary RGW. We see that in gap- RGW, the introduced gap acts as a PMC wall which divides the wave propagation path into two parts along both sides of the gap. As a result, a single gap-RGW can be interpreted as two separate RGWs with shorter widths. So, as RGW is a TEM-based structure, it is expected that the phase velocity at both sides of the gap should be almost the same. In Fig. 3, it can be observed that in the gap- RGW, the guided wavelength and consequently the phase velocity at both sides of the gap is almost equal and remains unchanged compared to that in ordinary RGW for both symmetric and asymmetric geometries. However, in the asymmetric case, we see a slight change in the phase velocity as a QTEM wave instead of an ideal TEM wave propagating inside the structure. In the asymmetric case, the phase velocity in the narrower path is slightly more than that in the wider one. In Fig. 3, we also see that the gap-RGW in its asymmetric form presents a power dividing feature as the wider side can handle more power than the other side. In Fig. 4, the E-field amplitude distribution over the waveguide cross section for the three cases shown in Fig. 3, is depicted. Here also it can be observed that for the symmetric gap-RGW, the power is equally divided between the two halves while in the asymmetric case, we see that more power is propagating inside the wider section. The power ratio of the two sides depends on the width ratio of the two RGWs created at both sides of the gap.

The power dividing feature of gap-RGW can be applied to provide amplitude tapering making use of an array of gap-RGWs. We have used this idea in an H-plane RGW horn (see Fig. 1) to properly control the amplitude tapering across the antenna aperture so that very low values of SLL can be obtained.

### Amplitude taper control in the horn

**Figure 5:**
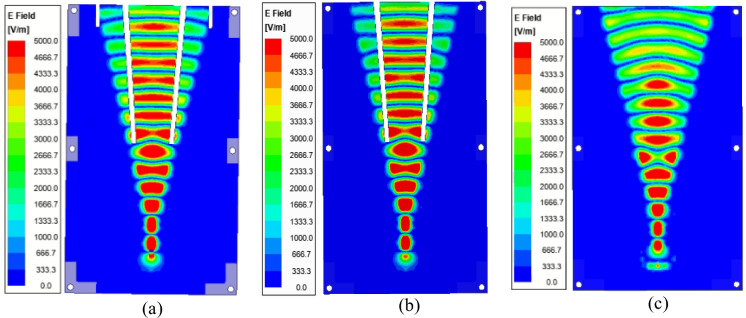
E-field distribution inside (**a**) the proposed gap-RGW horn (long Gaps + short Gaps) (**b**) the proposed gap-RGW horn (long Gaps only) and (**c**) its ordinary RGW counterpart at 14 GHz.

Here, as shown in Fig. 1, by introducing two pairs of long flared gaps to the RGW horn, two gap-RGWs are developed along the wave path inside the flared part of the horn. In this way, the same as the fork shaped horn presented in^28^, the wave propagation path inside the horn area is divided into three parts with unequal cross sections. As the middle path is wider, more power is expected to pass through it which leads to amplitude tapering at the antenna aperture. Moreover, the middle part (i.e. the wider section) introduces lower phase velocity as well as shorter physical length which can lead to lower phase error across the aperture. In Fig. 5, the field distribution inside the proposed gap-RGW horn is shown at 14 GHz and compared to that of the ordinary RGW counterpart. It can be observed that with the proposed structure both phase uniformity and amplitude taper are significantly improved leading to enhanced radiation performance.

**Figure 6:**
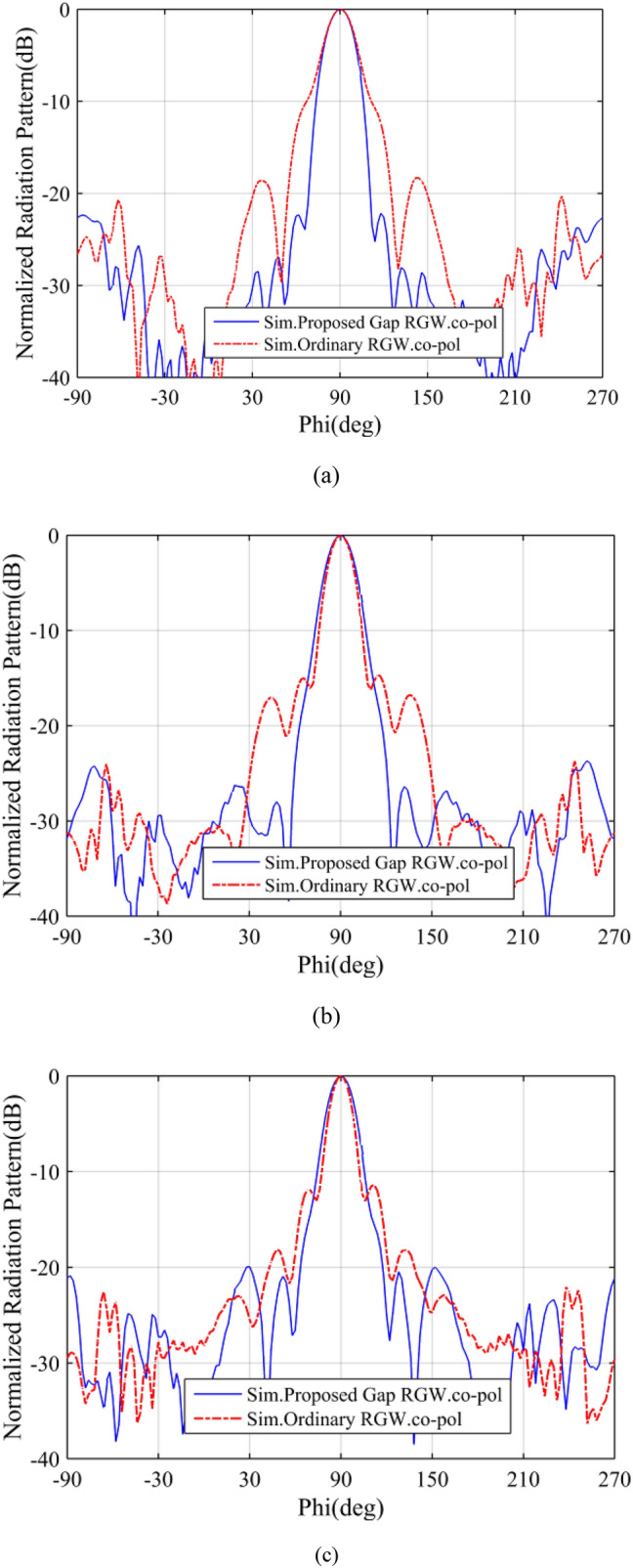
Simulated H-plane radiation patterns of the proposed gap-RGW horn and its ordinary RGW counterpart at (**a**) 13 GHz (**b**) 14.5 GHz and (**c**) 16 GHz.

**Figure 7:**
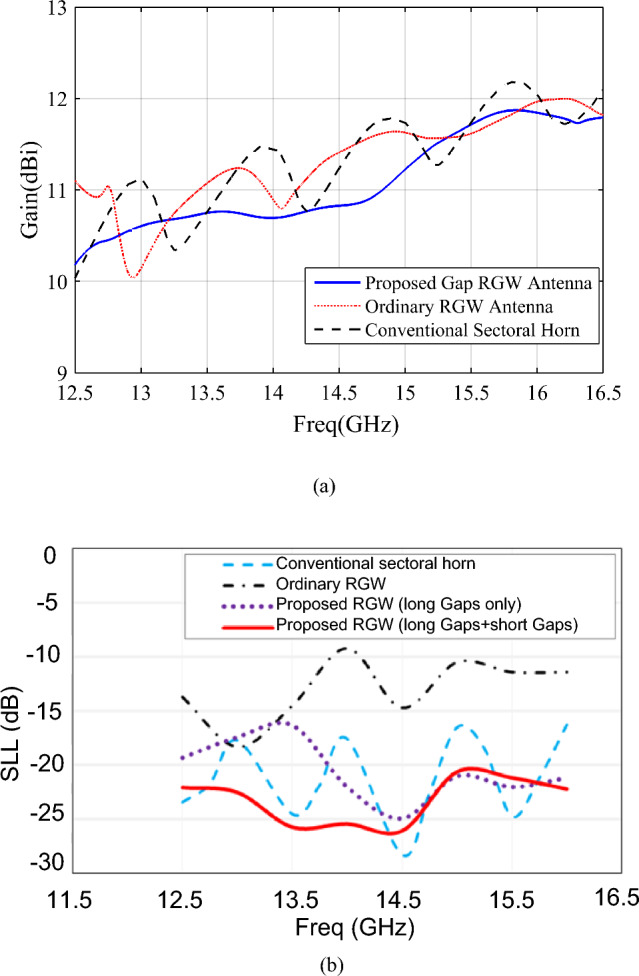
Comparison between the radiation performance of the proposed gap RGW-horn and its ordinary RGW and conventional sectoral horn counterparts, (**a**) gain (**b**) SLL in H-plane. W3 = 3 mm, W4 = 2.5 mm, L2 = 83.5 mm, L5 = 13.5 mm.

In Figs. 6 and 7a, the H-plane radiation patterns and the realized gains of the proposed horn are compared with those of the ordinary RGW counterpart over the 12–16 GHz frequency band. We see that the proposed horn presents much lower SLL along with quite the same gain, with a lower than 1 dB difference, over the most of desired bandwidth. It is well known that as SLL decreases, the directivity and consequently the gain decreases as well. However, here we see that despite the proposed structure presenting a significant reduction in SLL, its gain remains quite unchanged compared to the ordinary RGW counterpart which is due to the enhancement of phase uniformity at the proposed horn aperture.

To improve the proposed antenna radiation performance, as shown in Fig. 1, we modified the structure by introducing two extra short gaps with the length “L5” on the top plate at both sides of the long-flared gaps. For simplicity, these two short gaps are considered to be straight. In Fig. 7b, the SLL of the initial gap-RGW horn versus frequency is shown and compared with those of its modified version and the ordinary RGW counterpart. We see that with the modified version, the SLL of below -20 dB over the whole desired bandwidth is obtained which shows around 10 dB SLL improvement compared to the ordinary RGW horn over most of the bandwidth. In Fig. 7 the gain and SLL of the proposed horn are also compared with those of the same-sized conventional sectoral horn and we see that the proposed horn presents a lower gain (less than 1 dB) and lower SLL over most of the operating bandwidth.

### Parametric study of the gaps

**Figure 8:**
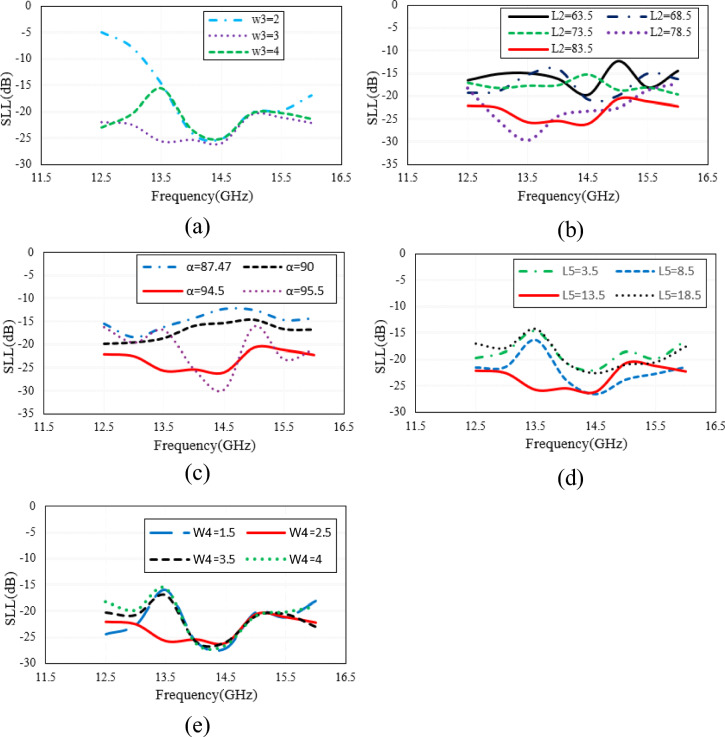
The effect of important geometrical parameters on the SLL of the proposed H-plane (**a**) W3 with W4 = 2.5 mm, L2 = 83.5 mm, L5 = 13.5 mm (**b**) L2 variation with W3 = 3 mm, W4 = 2.5 mm, L5 = 13.5 mm (**c**) α variation with W3 = 3 mm, W4 = 2.5 mm, L2 = 83.5 mm, L5 = 13.5 mm (**d**) L5 variation with W3 = 3 mm, W4 = 2.5 mm, L2 = 83.5 mm (**e**) W4 variation with W3 = 3 mm, L2 = 83.5 mm, L4 = 13.5 mm.

**Figure 9:**
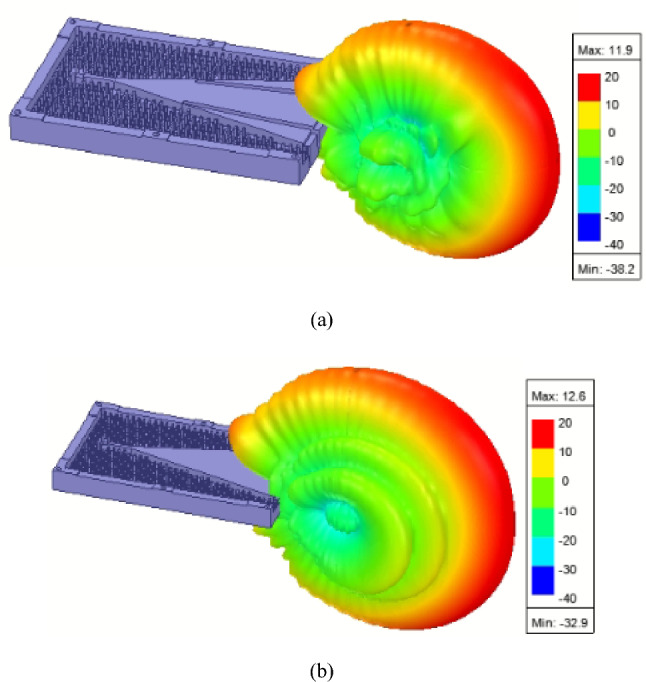
3D radiation pattern of (**a**) the proposed gap-RGW H-plane horn and (**b**) its ordinary RGW counterpart at 14.5 GHz.

In the proposed structure, the geometrical parameters of the introduced gaps including “L2”, “L5”, “W3” and “W4” affect both phase error and amplitude taper. So, a parametric study is done by full wave simulation to show how each parameter can affect the SLL. The results are illustrated in Fig. 8. We know that as the surface current vectors of the TEM wave propagating inside the horn, are not cut by the introduced gaps, no parasitic radiation through the gaps has occurred if the gap widths “W3” and “W4” are selected small enough. On the other hand, if the gap width is selected too small (the case happened in Fig. 8a for W3 = 2 mm at lower frequencies), then it will not be sensed by the propagating wave, and as a result, no gap-RGW is provided which means that no amplitude tapering and consequently no SLL reduction is achieved. So, the initial value of around λ/10, where λ is the free space wavelength, can be considered for the gap width and then it should be fine-tuned using full wave simulation. It should also be noted that the flare angle of long gaps “α” has to be properly adjusted to achieve the best performance in terms of both directivity and SLL. The initial value of “α” can be considered as 90̊ representing the straight long gaps. Comparing the results shown in Fig. 8a,b,c with those presented in Fig. 8d,e, we can conclude that the long gaps mainly control the amplitude taper and consequently the SLL, however the short gaps, as mentioned before, are used to optimize it. In Fig. 9, the 3D radiation pattern of the proposed horn is shown and compared with that of the ordinary RGW counterpart. A comparison of the two 3D patterns reveals that in the proposed horn, no considerable parasitic radiation is caused by the introduced gaps. It can also be observed that in the proposed structure, the SLL is significantly reduced in all directions.

## Measurement results and discussion

**Figure 10:**
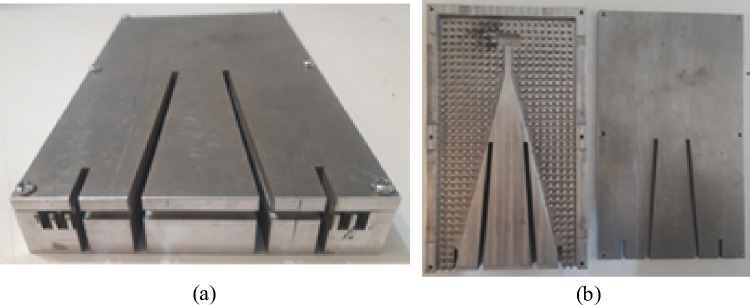
Fabricated prototype (**a**) front view (**b**) bottom and top layers.

**Figure 11:**
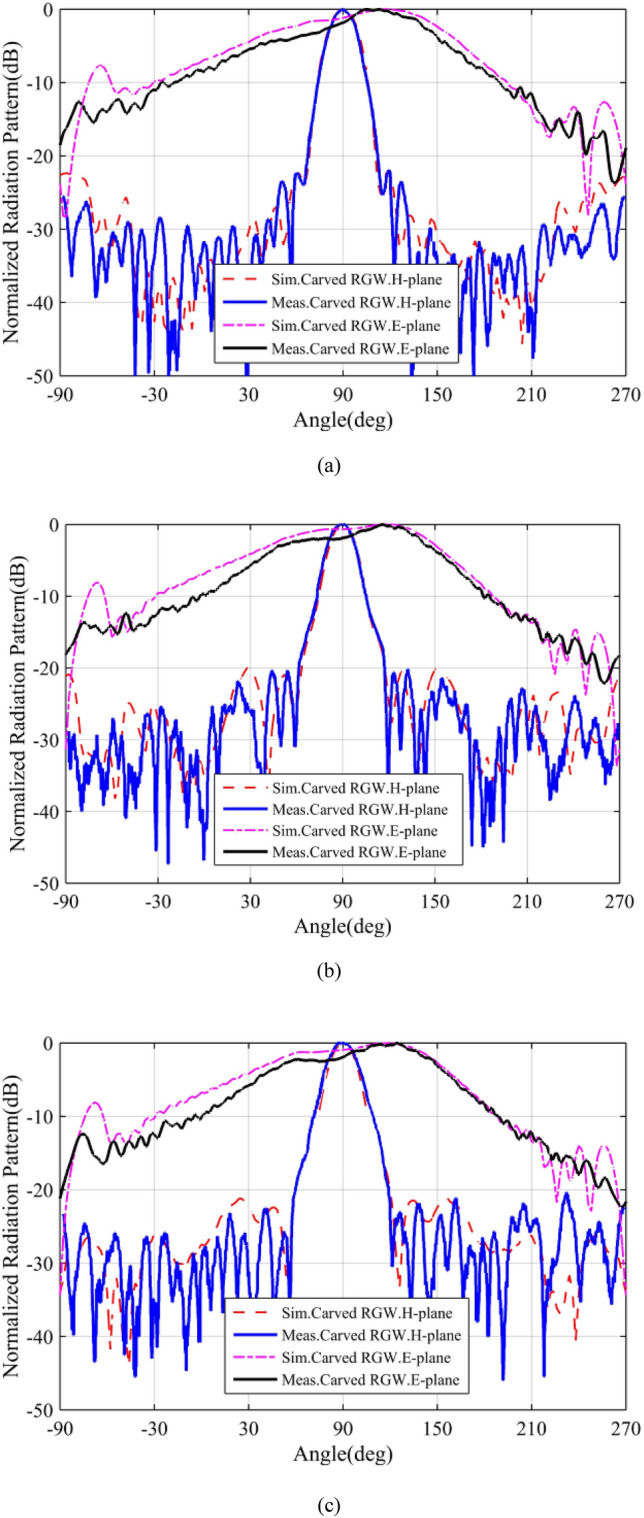
H- & E-plane radiation patterns of the designed antenna at (**a**) 13 GHz (**b**) 15 GHz (**c**) 16 GHz.

**Figure 12:**
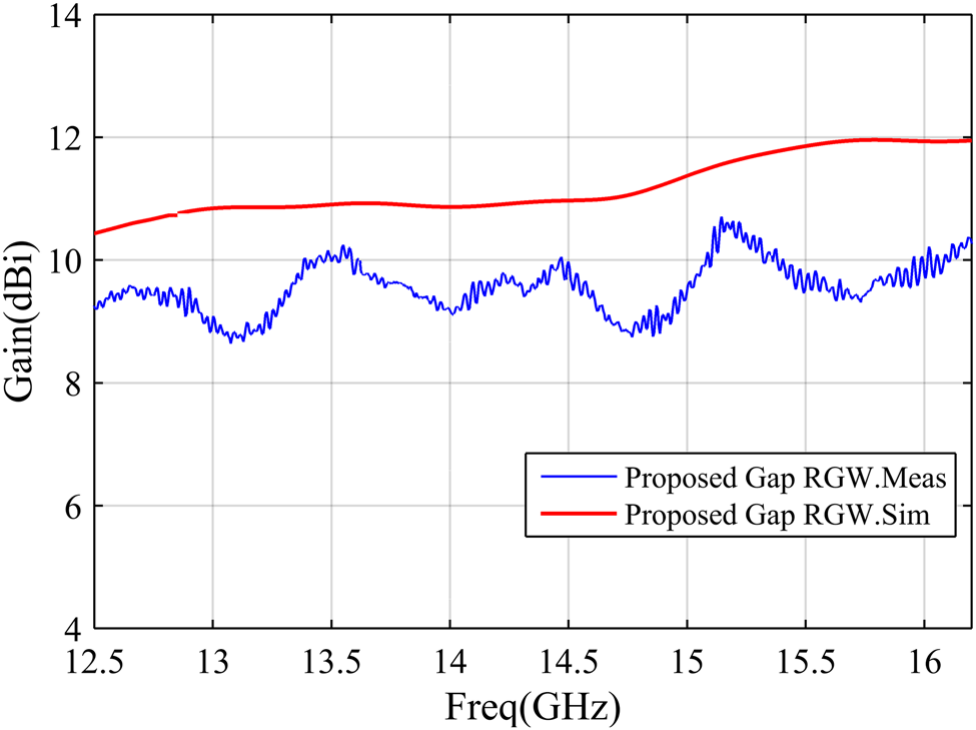
Simulated and measured gains of the designed antenna.

**Figure 13:**
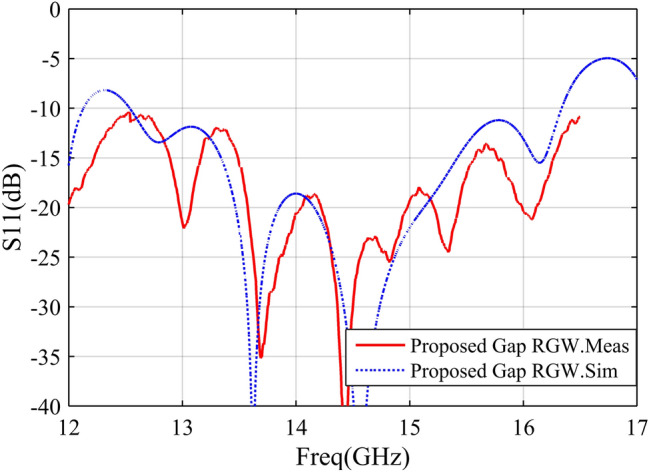
Simulated and measured reflection coefficients of the designed antenna.

According to the descriptions presented in the previous sections, a sample of the proposed antenna is designed to operate in the Ku band. The designed prototype with the dimensions listed in Table 1, is fabricated (see Fig. 10) and measured in an anechoic chamber. The simulation and measurement results of the designed antenna are illustrated and compared through Figs. 11, 12, 13. In Fig. 11, the H-&E-plane radiation patterns at three frequencies over the antenna operating bandwidth are shown and good agreement between simulated and measured results can be observed. Both simulation and measurement results shown in Fig. 11 confirm the SLL below − 20 dB in the H-plane over the most of operating bandwidth. In Fig. 11, we see that the E-plane radiation pattern is slightly deflected from the 90° angle which is due to the asymmetry of antenna aperture in E-plane (z–y plane). The antenna gain and reflection coefficient versus frequency are depicted in Figs. 12 and 13 respectively and we see that both measured and simulated results represent the curves with quite the same trends. As depicted in Fig. 12, the measured gain is 8.6–10.7 dBi over the 12–16.2 GHz frequency band which shows a 3 dB gain bandwidth of 30%. However, the simulated result shows a gain of 10–12 dBi over the same bandwidth. The discrepancy between the simulated and measured gains may be due to losses and inaccuracies associated with our available measurement setup which are not considered in the simulation. In Fig. 13, both simulation and measurement results show proper impedance matching (S11 < − 10 dB) over 12.5–16.2 GHz, i. e. the impedance bandwidth of 25%.

**Table 1:** Geometrical parameters of the designed antenna.

Parameter	Value (mm)	Parameter	Value (mm)
a	4.5	L	128.5
b	1.4	W1	31.17
g	1	W2	65
d	6	W3	3
L1	3.6	W4	2.5
L2	83.5	W5	3
L3	2.3	h1	2
L4	4.6	h2	4.8
L5	13.5

In Table 2, a comparison is provided between the proposed H-plane horn and some other GW H-plane horn antennas reported in the literature. We see that the antenna presented in this paper introduces the lowest SLL compared to all presented works except the one in^22^. However, the work presented in^22^ suffers from more fabrication complexity due to the use of a partially dielectric filled version of a gap waveguide.

**Table 2:** Comparison between the proposed H-plane horn and some reported H-plane antennas.

	Gain (dBi)	Size(λ_0_^2^)	Max SLL	Bandwidth (%)	Beam shape	Technology
16	12–14	4.9 × 8.2	− 10 dB	30	Fan beam	RGW
17	13.6–15.6	6.12 × 8.15	− 10 dB	42.6	Fan beam	RGW
19	8.5–12	9.25 × 5.5	− 16 dB	38	Fan beam	GGW
20	11.8–15.5	12.35 × 7.15	− 14 dB	34	Fan beam	RGW
21	14–17	4.5 × 4.95	− 10 dB	49	Pencil beam	Partially dielectric filled GGW
22	7.7–11.5	4.9 × 8.2	− 24 dB	28	Fan beam	Partially dielectric filled RGW
23	16.35–18.9	9.1 × 3.03	− 10 dB	42	Fan beam	GGW
24	12–13.55	4.95 × 6.42	− 8 dB	12	fan beam	GGW
25	3–9	4.7 × 2.14	− 10 dB	8	Fan beam	E-SIGW
26	11.3	2.25 × 3.33	− 6 dB	4	Fan beam	RGW
27	13.5–15.9	4.9 × 3.5	− 18 dB	17.3	Fan beam	GGW
This work	8.6–10.7	4.9 × 8.2	− 20 dB	25	Fan beam	Gap-RGW

## Conclusion

This paper presents a new topology for H-plane horns using gap waveguide technology. The proposed structure takes advantage of very low SLL of below − 20 dB over the 12–16.2 GHz frequency band (30%). The new gap-RGW concept which is presented in this paper for the first time is applied to control the amplitude taper across the horn aperture and consequently the SLL. We also see that this technique can enhance the phase uniformity across the antenna aperture as well. The proposed structure is very simple and shows proper radiation performance including flat gain response, low SLL, and proper impedance matching over quite wide bandwidth.

## Data Availability

Data sets generated during the current study are available from the corresponding author upon reasonable request. Please, contact (m.mohammadpour@shirazu.ac.ir).
